# FLI-1 Flightless-1 and LET-60 Ras control germ line morphogenesis in *C. elegans*

**DOI:** 10.1186/1471-213X-8-54

**Published:** 2008-05-16

**Authors:** Jiamiao Lu, William L Dentler, Erik A Lundquist

**Affiliations:** 1 Department of Molecular Biosciences, The University of Kansas, 1200 Sunnyside Avenue, Lawrence, KS 66045, USA; 2 Current address : Stanford University School of Medicine, Departments of Pediatrics and Genetics, 300 Pasteur Drive, Room H310, Stanford, CA 94305-5208, USA

## Abstract

**Background:**

In the *C. elegans *germ line, syncytial germ line nuclei are arranged at the cortex of the germ line as they exit mitosis and enter meiosis, forming a nucleus-free core of germ line cytoplasm called the rachis. Molecular mechanisms of rachis formation and germ line organization are not well understood.

**Results:**

Mutations in the *fli-1 *gene disrupt rachis organization without affecting meiotic differentiation, a phenotype in *C. elegans *referred to here as the germ line morphogenesis (Glm) phenotype. In *fli-1 *mutants, chains of meiotic germ nuclei spanned the rachis and were partially enveloped by invaginations of germ line plasma membrane, similar to nuclei at the cortex. Extensions of the somatic sheath cells that surround the germ line protruded deep inside the rachis and were associated with displaced nuclei in *fli-1 *mutants. *fli-1 *encodes a molecule with leucine-rich repeats and gelsolin repeats similar to Drosophila flightless 1 and human Fliih, which have been shown to act as cytoplasmic actin regulators as well as nuclear transcriptional regulators. Mutations in *let-60 Ras*, previously implicated in germ line development, were found to cause the Glm phenotype. Constitutively-active LET-60 partially rescued the *fli-1 *Glm phenotype, suggesting that LET-60 Ras and FLI-1 might act together to control germ line morphogenesis.

**Conclusion:**

FLI-1 controls germ line morphogenesis and rachis organization, a process about which little is known at the molecular level. The LET-60 Ras GTPase might act with FLI-1 to control germ line morphogenesis.

## Background

The *C. elegans *gonad is a bi-lobed organ composed of the germ line and somatic distal tip cells and sheath cells that partially envelop the germ line [1,2]. The distal half of the germ line is a syncytium, with multiple germ nuclei sharing a common cytoplasm. At the distal tip of the two gonad arms, germ line stem cells interact with the distal tip cell, which maintains them in a mitotic stem cell fate (the mitotic zone) [1,3]. As the nuclei proceed proximally down the germ line and lose contact with the distal tip cell niche, they exit mitosis and begin meiotic differentiation (the transition zone).

When the nuclei enter meiosis and arrest at pachytene in the meiotic zone, they are associated with the germ line cortex, resulting in a nucleus-free inner core of cytoplasm called the rachis [4,1,2]. Germ line plasma membrane invaginates between the nuclei to partially enclose them, forming a characteristic "T" structure of plasma membrane surrounding the meiotic germ nuclei [4,5]. Somatic sheath cells partially envelop the germ line and extend filopodia over bare regions, but do not extend protrusions deeply between germ line plasma membrane invaginations [4]. As pachytene nuclei reach the flexure, or bend, of the gonad arm, individual meiotic nuclei enter diakinesis and become completely enclosed by plasma membrane to complete oogenesis. Oocytes are fertilized as they move proximally through the spermatheca.

In recent years, genes and signals that control mitotic stem cell character and meiotic differentiation have been identified [6,2]. LAG-2 Delta in the distal tip and GLP-1 Notch in the germ line are required to maintain the mitotic stem cell fate in the distal tip cell niche and to repress the translation of meiotic differentiation factors [7-10]. As germ nuclei leave the niche, the meiotic differentiation factors GLD-1, 2, and 3 and NOS-1 promote meiosis and repress GLP-1 translation and the mitotic fate [11-15]. The transition zone contains a mix of mitotic and meiotic nuclei that reorganize to the cortex to form the rachis. Meiotic nuclei at the cortex arrest in pachytene until they reach the gonad flexure, where meiosis resumes and oogenesis begins. Ras/Map kinase signaling, including LET-60 Ras, is required for progression through pachytene and entry into diakinesis [16,17]. While much is known about meiotic specification, less is known about the molecular mechanisms that control rachis organization in the meiotic zone, although Ras signaling is likely involved, as mutations in *let-60 Ras *and *mpk-1 Erk *cause disorganization of the pachytene region of the germline.

Described here are initial studies showing that the *fli-1 *gene perturbs rachis formation without affecting meiotic progression. In *fli-1 *mutants, chains of germ nuclei were observed in the rachis of the meiotic zone, and ultrastructural analysis revealed that these nuclei remained associated with germ line plasma membrane. Furthermore, extensions of the sheath cells protruded into the rachis between these misplaced nuclei. This phenotype is referred to here as a germ line morphogenesis defect (the Glm phenotype). No defects in mitotic or meiotic specification were observed in the misplaced nuclei or in any germ nuclei in *fli-1 *mutants.

The *fli-1 *locus was identified and found to encode a molecule with N-terminal leucine-rich repeats (LRRs) and 5 C-terminal gelsolin repeats, similar to the Drosophila and human Flightless 1 molecules [18,19]. *C. elegans *FLI-1 can bind to and sever actin filaments [20], and a *fli-1 *mutation caused defects in a variety of tissues, including germ line organization defects [21]. Human Flightless 1 (fliih), along with a monomer of G-actin, is a component of a transcriptional coactivator complex that acts with nuclear hormone receptors and β-catenin/TCF LEF [22,23]. In Drosophila, *Flightless 1 *mutants display defects in flight muscle development as well as defects in nuclear organization and cellularization in the syncytial blastoderm [19]. Thus, Flightless 1 molecules might have distinct roles in the cytoplasm and nucleus, possibly organizing the actin cytoskeleton in the former and modulating transcription in the latter.

The LET-60 Ras molecule has been shown to control meiotic progression from pachytene to diakinesis, and *let-60 *mutations were found to have a germ line organization defect [16]. Data presented here show that *let-60 Ras *has a Glm phenotype similar to *fli-1*, and that *let-60 Ras *and *fli-1 *interact genetically in germ line morphogenesis. Thus, FLI-1 and LET-60 Ras might act together to control germ line organization and rachis formation during meiotic differentiation in the *C. elegans *germline.

## Results

### *fli-1(ky535) *affects germ line morphogenesis

The *ky535 *mutation was isolated in a synthetic lethal screen to identify molecules that act in parallel to the actin-binding protein UNC-115 abLIM [24]. UNC-115 and FLI-1 likely have roles in pharyngeal function underlying the synthetic lethal phenotype. Pharyngeal pumping is severely reduced in *unc-115; fli-1 *double mutants, and double mutants arrest in the L1 larval stage consistent with a feeding defect (data not shown).

**Figure 1:**
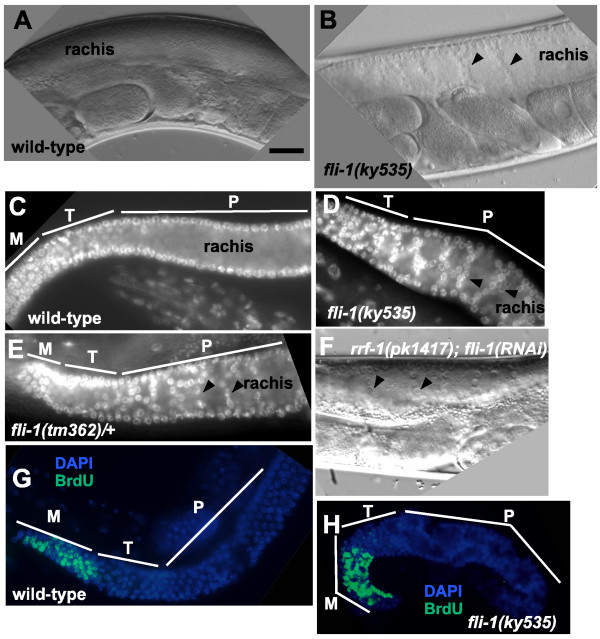
***fli-1 *mutants display germ line morphogenesis defects (the Glm phenotype)**. In all images, the distal tip of the gonad is to the left. In images (C), (D), (E), (G), and (H), the approximate extents of mitotic zones (M), transition zones (T), and meiotic pachytene zones (P) are indicated. (A) and (B) Differential Interference Contrast (DIC) images of wild-type and *fli-1(ky535) *gonads. A wild-type gonad had a germ nucleus-free rachis in the meiotic zone whereas a *fli-1(ky535) *animal displayed chains of nuclei crossing the rachis (arrowheads in (B)). (C-E). Epifluorescence images of DAPI-stained gonads from wild-type, *fli-1(ky535)*, and *fli-1(tm362)/+ *animals. Wild-type shows a nucleus-free rachis, whereas *fli-1(ky535) *and *fli-1(tm362)/+ *displayed chains of nuclei crossing the rachis (arrowheads). (F) DIC image of a gonad from an *rrf-1 *mutant animal subject to *fli-1 *RNAi (*rrf-1 *animals are defective for somatic RNAi but not germ line RNAi). Arrowheads indicate nuclei in the rachis. (G) and (H) Gonads from wild-type and *fli-1(ky535) *fed BrdU-containing bacteria for 5 minutes and fixed and stained with DAPI and anti-BrdU antibody. Nuclei in the mitotic zone of both wild-type and *fli-1(ky535) *accumulated BrdU. No BrdU-positive nuclei were seen in the meiotic pachytene regions, including the misplaced nuclei in *fli-1(ky535*). The scale bar in (A) = 10 μm for (A-H).

**Figure 2:**
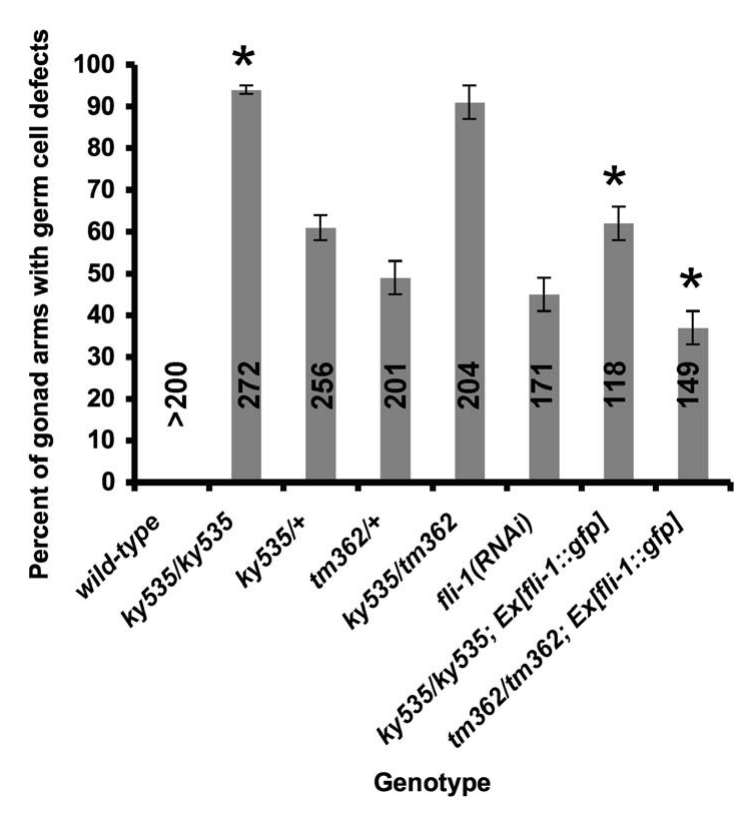
**Quantitation of the Glm phenotype in *fli-1 *mutants**. Genotypes are indicated along the y axis, and percent of gonad arms displaying the Glm phenotype is the x axis. For each genotype, the number of gonad arms scored is indicated inside the bar, and the standard error of the proportion is shown as error bars. Asterisks indicate that the differences between genotypes are significant (p < 0.001; t-test and Fisher's Exact analysis).

Alone, *fli-1(ky535) *animals were viable and fertile and displayed a slightly Dumpy (Dpy) body morphology. When observed using differential interference contrast (DIC) microscopy, germ line nuclei were observed in the rachis of the meiotic zone (compare Figures 1A and 1C to Figures 1B and 1D). In most cases, chains of apparently connected nuclei spanned the rachis. Misplaced germ cells in the rachis were observed as soon as the rachis was evident in mid-to-late L4 larval animals (data not shown). This phenotype is referred to here as the germ line morphogenesis (Glm) phenotype. In *fli-1(ky535)*, 94% of gonad arms displayed the Glm phenotype (Figure 2). The Glm phenotype was never observed in wild-type animals.

### Transition from mitosis to meiosis is not disrupted in *fli-1(ky535) *mutant germ nuclei

The misplaced nuclei in the rachis of the meiotic zone in *fli-1 *might have been due to disruption in the transition of nuclei from mitosis to meiosis. A BrdU incorporation was used to assay nuclei undergoing DNA synthesis in the germ line (e.g. those that have undergone mitosis or S phase of meiosis I) (see Methods) [25]. After 10 minutes of exposure to BrdU, wild-type animals displayed BrdU-positive nuclei in the distal mitotic zone (Figure 1G). *fli-1(ky535)*-mutant gonads displayed a similar BrdU incorporation profile (Figure 1H), and nuclei in the rachis of the meiotic zone did not incorporate BrdU. A 30-minute exposure to BrdU also resulted in no apparent differences between wild-type and *fli-1(ky535) *(data not shown). In sum, no differences in BrdU incorporation were detected between wild-type and *fli-1(ky535)*, suggesting that misplaced nuclei in the rachis of the meiotic zone of *fli-1(ky535) *were not undergoing mitotic divisions, and that normal meiotic progression was not affected (e.g. meiosis I was not delayed). DAPI staining to assay nuclear morphology showed that misplaced nuclei in the rachis of the meiotic zone of *fli-1(ky535) *animals displayed a meiotic pachytene morphology; the pachytene chromosomes were individually visible with a "bowl of spaghetti" appearance (Figure 1D) [2].

**Figure 3:**
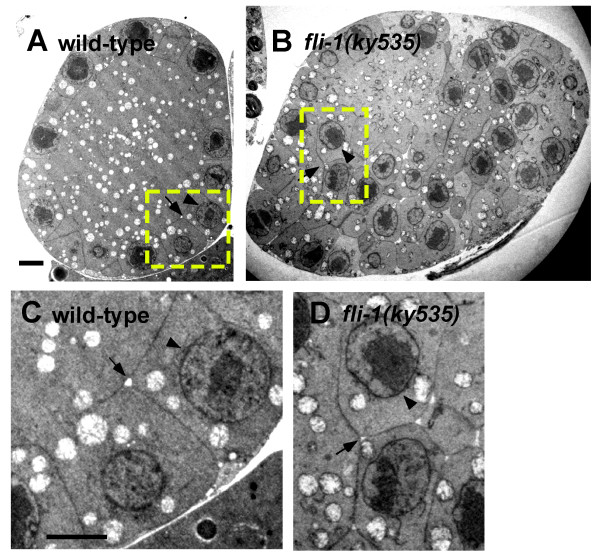
**Misplaced nuclei in *fli-1(ky535) *are partially surrounded by germ line plasma membrane**. Shown are transmission electron microscopy cross-sections of gonads from wild-type and *fli-1(ky535)*. (A) A cross-section through the meiotic pachytene zone of wild-type. The nuclei are arranged at the cortex (the arrowhead indicates a nucleus) and are partially surrounded by germ line plasma membrane, forming the "T" structure (arrow). (B) Cross section through the meiotic pachytene zone of a *fli-1(ky535) *mutant. Misplaced nuclei are apparent (arrow), and misplaced nuclei are surrounded by germ line plasma membrane in a manner similar to nuclei at the cortex (internal "T" structure-like membrane organization is indicated by the arrow). (C and D) Magnification of regions in the dashed boxes in (A) and (B). The arrowheads indicate nuclei and the arrows indicate germ line plasma membrane. Scale bars = 2 μm for all micrographs.

In transmission electron microscopic (TEM) cross-sections, nuclei in the meiotic zone in *fli-1(ky535)*, including misplaced nuclei, were of roughly the same size and shape as those in wild-type (Figure 3; 2.9 ± 0.06 μm diameter for *fli-1(ky535) *and 3.0 ± 0.6 μm diameter for wild-type). The misplaced nuclei in *fli-1(ky535) *had a meiotic appearance (Figure 3). Meiotic nuclei appear round and regular as those seen in Figure 3C and 3D, whereas mitotic nuclear membranes have an irregular, "wavy" appearance. These lines of evidence indicate that the transition from mitosis to meiosis is unaffected in *fli-1(ky535) *mutant germ nuclei.

The previously-published *fli-1(bp130) *allele caused defects in oocyte production and brood size [21]. Brood size of *fli-1(ky535) *was comparable to that of wild-type (an average of 272 progeny for *fli-1(ky535) *compared to 319 for wild type; t-test p = 0.11). Possibly, *bp130 *is a stronger allele of *fli-1 *than is *ky535 *and affects oocyte production more strongly than *ky535*.

### Germ line plasma membrane partially surrounded misplaced germ nuclei in *fli-1(ky535)*

**Figure 4:**
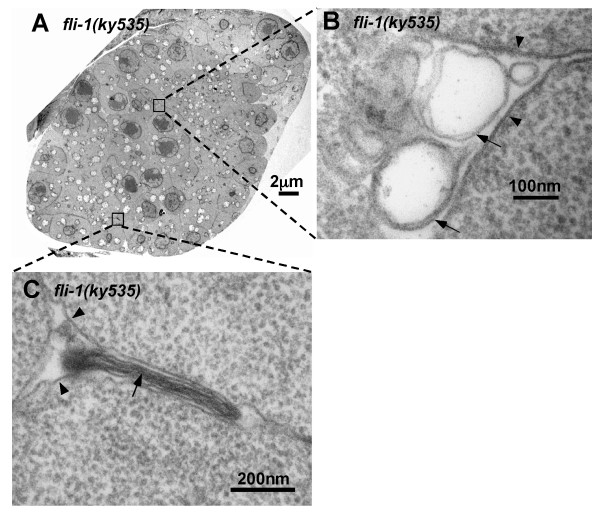
**Membrane-like structures are present between misplaced germ nuclei in *fli-1(ky535)***. Shown are transmission electron microscope cross-sections in the meiotic pachytene zone of *fli-1(ky535) *mutants. (A) *fli-1(ky535) *displays misplaced nuclei and associated germ line plasma membrane. (B and C) Higher-magnification views of regions between the germ line plasma membrane (arrowheads) surrounding misplaced nuclei. Arrows indicate membrane-like structures in the interstices between germ line plasma membrane. An electron-dense laminar structure is shown in (C).

TEM analysis revealed that wild-type meiotic zone nuclei were near the cortex. The germ line plasma membrane protruded between and partially enveloped each nucleus, forming a characteristic "T" shaped membrane described above and elsewhere (Figure 3A and 3C) [4,5]. In TEM cross-sections of meiotic regions of *fli-1(ky535)*, germ line plasma membrane was clearly associated with each misplaced nucleus in the rachis, suggesting that germ line plasma membrane invaginated to partially enclose misplaced germ nuclei (Figure 3B and 3D and Figure 4). A similar phenotype was observed in cross-sections of animals heterozygous for a deletion of the *fli-1 *locus called *tm362 *(data not shown). No defects in the organization of the distal mitotic zone were observed in cross sections of *fli-1(ky535) *(e.g. the germ cell arrangement resembled wild-type and distal tip cell filopodia between germ cells was observed). While the shape and diameter of wild-type distal meiotic gonads was relatively uniform (a diameter range of 16–23 μm, n = 10), *fli-1 *gonads were often of irregular diameter (a range of 12–33 μm, n = 10) and irregular shape (compare Figures 3A with Figure 3B and 4A).

### Sheath cell extensions were associated with misplaced nuclei in the rachis in *fli-1(ky535)*

The plasma membrane surrounding interior nuclei in *fli-1(ky535) *formed gaps between nuclei similar to the gaps formed by plasma membrane invagination around cortical nuclei (Figure 3B and 3D and Figure 4). In *fli-1(ky535) *mutants, additional membranes were frequently observed occupying these interior gaps formed by invaginated germ line plasma membrane (Figure 4B and 4C). Less frequently, electron-dense laminar structures were present in the interior gaps (Figure 4C). Cross sections of heterozygous *fli-1(tm362)/+ *deletion animals showed a similar phenotype (data not shown).

The nature of these membrane-like structures between misplaced germ cells observed by TEM was unclear. The germ line is partially surrounded by the somatic sheath cells, which extend filopodia across the bare regions of the germline not covered by the cell body [4]. Sheath cell protrusions occupy gaps between nuclei formed by germ line plasma membrane invagination. In wild-type, sheath cell protrusions do not extend deeply between nuclei but rather stay near the cortex [4]. Possibly, the membrane-like structures between misplaced germ nuclei in *fli-1 *mutants were somatic sheath cell extensions.

**Figure 5:**
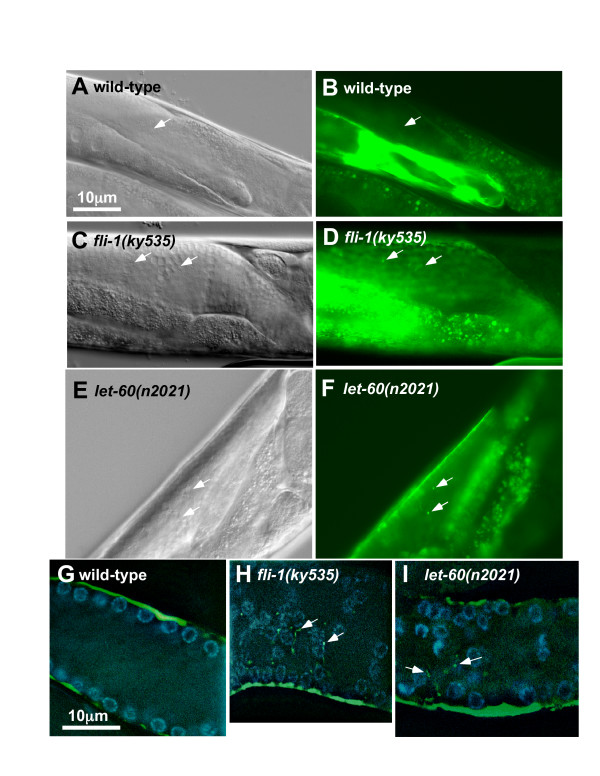
**Sheath cells projections extend into the rachis in *fli-1 *and *let-60***. (A-F) DIC and *lim-7::gfp *expression in wild-type, *fli-1(ky535)*, and *let-60(n2021) *(the same animals at the same focal planes are shown). (A), (B) No *lim-7::gfp *is observed in the rachis of wild-type (arrows). (C-F) Arrows point to displaced nuclei (in the DIC images) and associated fingers of *lim-7::gfp *expression in the rachis of these mutants. (G-I) Merged confocal micrographs of *lim-7::gfp *expression (green) and DAPI nuclear staining (blue) in dissected gonads. In *lfi-1(ky535) *and *let-60(n2021)*, extensions of *lim-7::gfp *are observed associated with displaced nuclei in the rachis (arrows). The scale bar in (A) applies to (A-F); the scale bar in (G) applies to (G-I).

To assay sheath cell morphology, a transgene consisting of the *lim-7 *promoter driving *gfp *expression was analyzed. *lim-7::gfp *is expressed in the sheath cells but not the germ line [4]. In wild-type harboring *lim-7::gfp*, no GFP fluorescence was detected in the rachis of the meiotic region (Figure 5A, B, and 5G), although GFP was detected at the surface of the germ line in a "honeycomb" pattern as previously described [4], due to the thin cytoplasm of regions of the sheath cells covering the germ nuclei.

**Figure 6:**
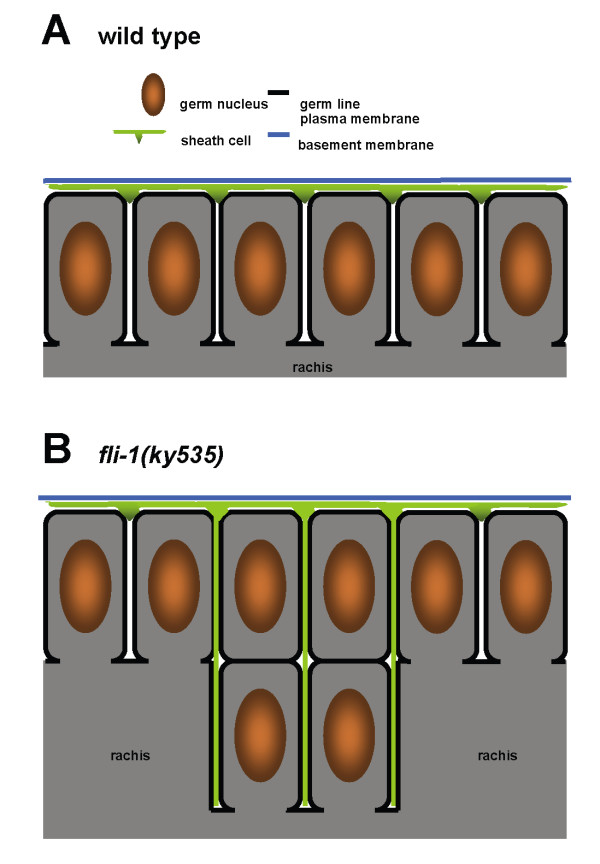
**A diagram of gonad organization in *fli-1***. Schematic diagram of the organization of the pachytene meiotic region of the germ cell in wild-type (A) and *fli-1 *mutants (B). In wild-type, germ cell plasma membrane protrudes between cortical nuclei and partially envelops the, forming the "T" structure. Sheath cells protrude superficially between nuclei. In *fli-1(ky535)*, nuclei in the rachis are enveloped with germ cell plasma membrane, and sheath cells protrude deeply into the rachis.

In *fli-1(ky535) *mutants, the cortical "honeycomb" pattern was observed, although it was often irregular and disorganized, suggesting cortical nucleus arrangement was disorganized. Fingers of GFP expression were observed protruding into the rachis and associating with misplaced germ nuclei (Figure 5C, D, and 5H). These protrusions were from the somatic sheath cells and not the distal tip cell, as a *lag-2::gfp *transgene, expressed only in the distal tip cell [9], did not show these patches in the rachis in *fli-1(ky535) *animals (data not shown). The TEM and *lim-7::gfp *results combined indicate that misplaced nuclei in the rachis were bounded by germ line plasma membrane, and extensions of the sheath cells protruded between the misplaced germ cells in the rachis (Figure 6 is a depiction of these results).

### FLI-1 encodes a molecule similar to Flightless 1/Fliih

**Figure 7:**
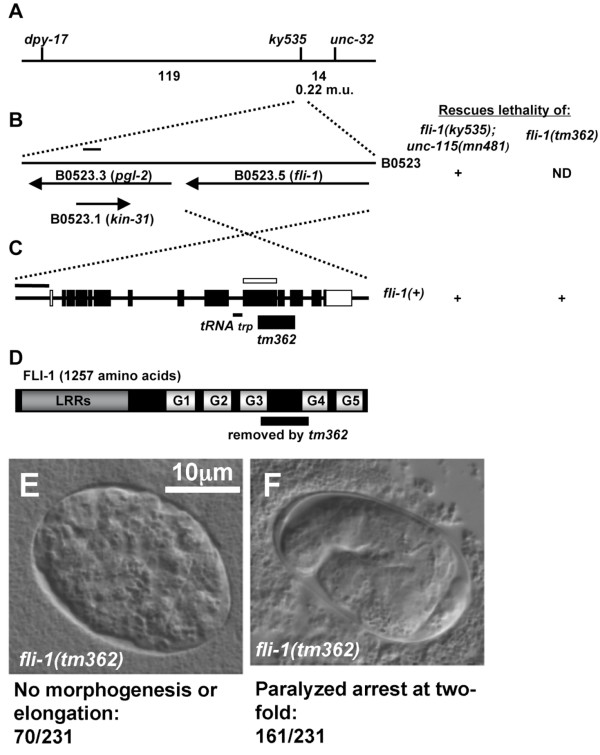
**The *fli-1 *locus**. (A) A genetic map of the *fli-1 *region. Numbers below the line indicate the number of recombination events between the loci in three-factor crosses. From *dpy-17(e1264) unc-32(e189)/fli-1(ky535) *trans-heterozygotes, 30/34 Dpy-non-Unc recombinants harbored *ky535 *and 10/99 Unc-non-Dpy recombinants harbored *ky535*. The estimated genetic distance between *unc-32 *and *ky535 *is 0.22 map units. (B) A diagram of the cosmid B0523. Genes on the cosmid are indicated as arrows. (C) The *fli-1 *gene. 5' is to the left. Black boxes indicate coding exons, and white boxes represent non-coding exons. The extent of the *tm362 *deletion is indicated below the line, as is the location of a Trp tRNA gene. The white box above the line indicates the region used in *fli-1 *RNAi experiments, and the black line represents 1 kb. The gene structure is derived from a *fli-1 *cDNA previously described (Genbank accession number U01183). (D) A diagram of the predicted FLI-1 polypeptide. The locations of the leucine-rich repeats (LRRs) and the five gelsolin-like repeats (G1–G5) are indicated. The deletion *fli-1(tm362) *removes coding region for the residues of FLI-1 indicated below the diagram. (E) A *fli-1(tm362) *homozygous embryo arrested before embryonic elongation. (F) A *fli-1(tm362) *homozygous mutant embryo arrested at the two-fold stage and showed a paralyzed arrest at two-fold (Pat) phenotype. The scale bar in (E) applies to (E) and (F).

The *ky535 *mutation was mapped genetically to linkage group III by standard linkage analysis with visible markers using synthetic lethality with *unc-115 *(data not shown). Three-factor mapping with *dpy-17 *and *unc-32 *using the Glm phenotype indicated that *ky535 *was close to and to the left of *unc-32 *(approximately 0.22 cM) (Figure 7A). The *fli-1 *gene (B0523.5 on Wormbase), which encodes an actin-binding protein of the Flightless 1/Fliih family, resides in this region of the genome (Figure 7B and 7C). The FLI-1 polypeptide is composed of N-terminal leucine-rich repeats (LRRs) and 5 C-terminal gelsolin-like domains (Figure 7D). A *fli-1 *cDNA was isolated in a previous study [26] (U01183 in Genbank). This transcript was used as the basis for Figure 7C and 7D. The cDNA is likely to contain the entire *fli-1 *coding region, as an in-frame stop codon is present 5 codons upstream of the presumed initiation methionine (data not shown). Furthermore, two independent *fli-1 *cDNAs were sequenced (yk48g9 and yk294b7, courtesy of Y. Kohara). While incomplete at the 5' ends, these cDNAs were identical in structure to the U01183 cDNA.

To test if the *fli-1 *gene is involved in germ line morphogenesis, RNA-mediated gene interference (RNAi) of *fli-1 *was performed. *fli-1(RNAi) *phenocopied the germ line morphogenesis defect of *ky535 *(Figure 1F and Figure 2). Furthermore, the cosmid B0523, which contains the *fli-1 *gene, rescued the synthetic lethality of *unc-115(mn481); fli-1(ky535) *animals harboring a transgene containing the cosmid (Figure 7B). The B0523 cosmid contains two other genes, B0523.1 and B0523.3. RNAi of these genes did not cause a Glm phenocopy (data not shown). *fli-1 *RNAi in both wild-type and *rrf-1(pk1417 *and *ok289) *backgrounds caused the Glm phenocopy (Figure 1F and Figure 2). *rrf-1 *mutations attenuate RNAi in somatic cells but do not apparently affect RNAi in the germ line [27].

A PCR-generated fragment of B0523 containing only the *fli-1 *gene and a tryptophan tRNA (Figure 7C, see Methods) rescued the synthetic lethality of *unc-115(mn481); fli-1(ky535) *mutants (Figure 7C). Furthermore, a *fli-1::gfp *full-length fusion transgene (see Methods) partially rescued the Glm phenotype of *fli-1(ky535) *animals (Figure 2) as well as the lethality of *unc-115(mn481); fli-1(ky535) *double mutants. The nucleotide sequence of the entire region included in the rescuing *fli-1(+) *transgene was determined from *ky535 *mutants. No nucleotide changes were detected in this region in three independent PCR amplifications of the *fli-1 *gene from *ky535 *genomic DNA. Possibly, *ky535 *is regulatory mutation outside of the region necessary for rescue, and transgenic *fli-1(+) *expression, which can often lead to overexpression, can overcome the *ky535 *mutation. A *fli-1 *transcript was detected by RT-PCR in *fli-1(ky535) *mutants (data not shown). As described below, the *fli-1 *locus is haploinsufficient for the Glm phenotype, indicating that lowering *fli-1 *gene dosage by as little as one-half can cause the Glm phenotype.

### *The fli-1(tm362) *deletion causes a germ line morphogenesis defect

To confirm that *fli-1 *controls germ line morphogenesis, a deletion in the *fli-1 *locus was analyzed (isolated and kindly provided by The National Bioresource Project for the Experimental Animal *C. elegans*, S. Mitani). The deletion, *tm362*, removed bases 10973 to 11931 relative to the cosmid B0523 (Genbank Accession number L07143) with breakpoints in coding exons 9 and 11 of *fli-1 *(Figure 7C). The out-of-frame *tm362 *deletion removed coding region encompassing parts of gelsolin domains 3 and 4 (Figure 7D).

*fli-1(tm362) *homozygotes from a heterozygous mother arrested during embryogenesis and failed to hatch. Of arrested embryos, 70% displayed the Pat phenotype (paralyzed and arrested at the two-fold stage of embryonic elongation) (Figure 7F). The Pat phenotype is characteristic of defects in body wall muscle function [28]. Indeed, *fli-1(ky535) *mutants displayed slightly disorganized myofilament lattice structure in body wall muscles (data not shown), suggesting that body wall muscle development was also affected by *fli-1(ky535)*. The remaining 30% of *tm362 *homozygous embryos arrested earlier in embryogenesis with severe defects in embryonic organization (Figure 7E). Defects in muscle organization and embryonic development in a *fli-1 *mutation have been described [21].

While homozygous *fli-1(tm362) *animals arrested in embryogenesis, heterozygous *tm362/+ *animals displayed the Glm phenotype similar to *ky535 *animals (49%; Figure 1E and Figure 2). TEM cross sections of *tm362/+ *heterozygotes were analyzed and found to have a similar ultrastructural defect as described for *fli-1(ky535) *(data not shown), including germ line plasma membrane and sheath cell invaginations around misplaced nuclei. These results suggest that *fli-1 *is haploinsufficient for the Glm phenotype. Indeed, heterozygous *ky535/+ *animals also displayed the Glm phenotype (60% compared to 94% for *ky535 *homozygotes; Figure 2). *Trans*-heterozygous *ky535/tm362 *animals were viable and had a severe Glm phenotype (91%; Figure 2), suggesting that *ky535 *and *tm362 *failed to complement for this phenotype. However, the additive effect of each heterozygote alone could explain this effect.

The lethality of *fli-1(tm362) *was rescued by the *fli-1(+) *transgene that also rescued the *unc-115(mn481); fli-1(ky535) *lethality (Figure 7C), and the Glm phenotype of rescued homozygous *fli-1(tm362) *animals was significantly less severe than *fli-1(ky535) *homozygotes (Figure 2). The Glm phenotype was likely due to *fli-1 *loss of function, as *fli-1 *RNAi caused the Glm phenocopy and the Glm phenotype of both *fli-1(ky535) *and *fli-1(tm362) *was rescued by transgenic *fli-1(+)*. Thus, the viable *fli-1(ky535) *allele might be hypomorphic and the lethal *fli-1(tm362) *allele might be null. If this is the case, *fli-1 *might be haploinsufficient for the Glm phenotype as heterozygotes displayed the Glm phenotype. It is also possible that either or both of the two *fli-1 *alleles are not simple loss-of-function alleles and thus cause a dominant Glm phenotype. Indeed, *fli-1(tm362) *was rescued more efficiently than *ky535 *by transgenic *fli-1(+) *(Figure 2), suggesting that *ky535 *might have some dominant character that is more difficult to rescue. In either case, the Glm defect is likely a loss-of-function phenotype of *fli-1 *as RNAi of *fli-1 *caused the Glm defect.

### Germ line actin organization in *fli-1(ky535) *mutants

FLI-1 can bind to and sever actin filaments [20], suggesting that it might modulate cytoskeletal organization. The effect of *fli-1(ky535) *on the actin cytoskeleton of the germ line was analyzed by staining with rhodamine-labeled phalloidin. Hermaphrodite somatic sheath cells contain much actin, which was difficult to distinguish from germ line actin. To circumvent this problem, male gonads, which lack sheath cells, were analyzed, although hermaphrodites showed a pattern consistent with that seen in males (data not shown).

**Figure 8:**
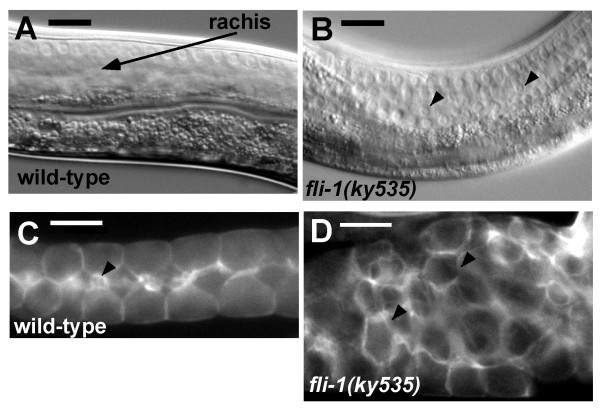
**Actin organization in the *fli-1(ky535) *male germ line**. (A) and (B). DIC images of the wild-type and *fli-1(ky535) *male germ lines in the meiotic zone. The arrow in (A) points to the rachis in wild-type, devoid of germ nuclei. The arrowheads in (B) point to misplaced nuclei in the rachis of the meiotic zone in *fli-1(ky535)*. (C) and (D) Rhodamine-phalloidin staining of male gonads. (C) Cortical actin surrounds each germ nucleus, and the arrowhead indicates increased intensity at presumptive "T" structures. The rachis is narrower in the male germ, but the rows of cortical nuclei can be clearly observed in this micrograph. (D) *In fli-1(ky535)*, cortical actin surrounds misplaced nuclei in the rachis, but actin organization *per se *is not obviously affected. Scale bars represent 10 μm.

*fli-1(ky535) *males displayed a Glm phenotype similar to hermaphrodites, as displaced nuclei were observed in the rachis of the single male gonad arm (Figure 8A and 8B). In the wild type male germ line, a cortical layer of phalloidin staining was associated with the germ line plasma membrane that surrounded each germ nucleus (Figure 8C). In *fli-1(ky535)*, nuclei at the cortex displayed an apparently normal actin organization. However, a cortical layer of actin was observed surrounding the misplaced nuclei in the rachis, apparently associated with the invaginated plasma membrane (Figure 8D). While actin was associated with misplaced nuclei in *fli-1(ky535)*, no obvious defects in the organization of the actin cytoskeleton *per se *were observed. The *fli-1(bp130) *allele caused defects in gonad actin organization [21] not seen in *fli-1(ky535)*. *fli-1(ky535) *might be a hypomorphic allele, and actin organization might not be affected to the extent observed in *bp130*.

### *fli-1 *is expressed in the gonad and in muscle

**Figure 9:**
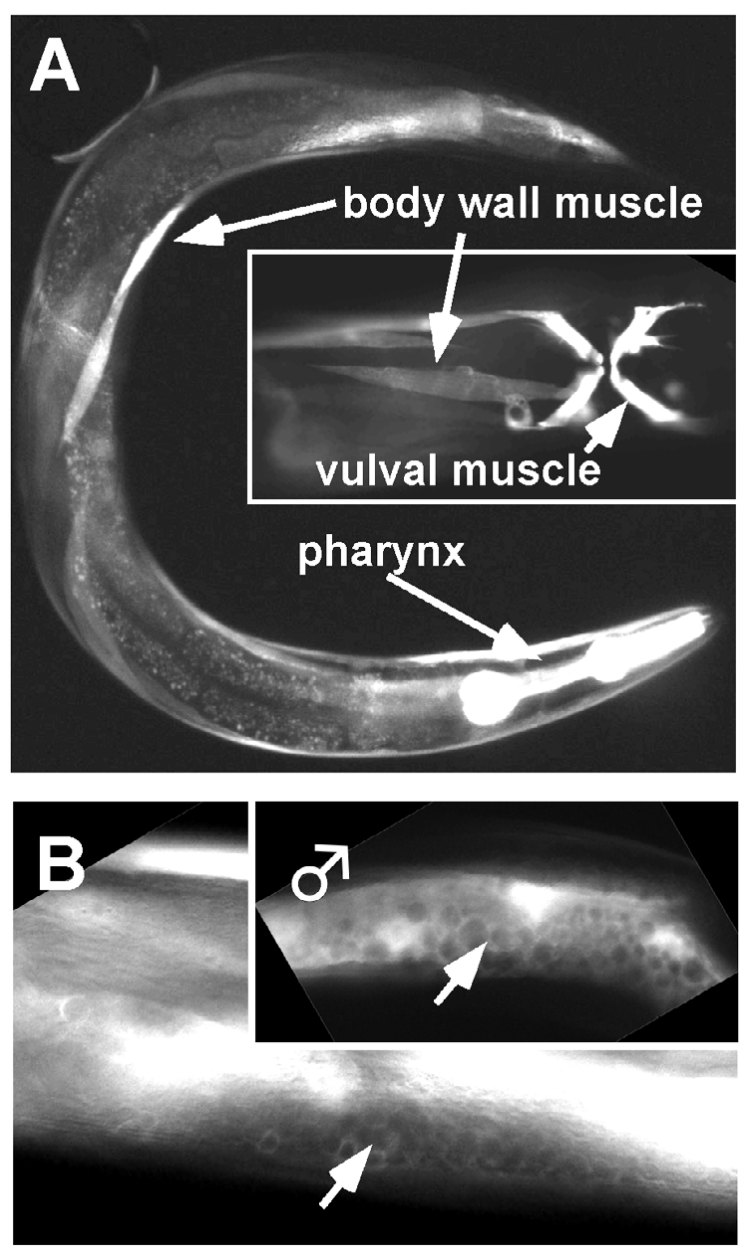
***fli-1::gfp *is expressed in the gonad and in muscle**. Shown are epifluorescence images of wild-type animals harboring a transgene consisting of the *fli-1 *promoter driving *gfp *expression. (A) Arrows point to *pfli-1::gfp *expression in body wall muscle, pharyngeal muscle, and vulval muscle (inset). (B) Arrows point to germ line expression in a hermaphrodite and a male (inset). The "honeycomb" pattern is due to exclusion of GFP from the nuclei of the germ line.

A transcriptional *fli-1promoter::gfp *reporter transgene was constructed that contained the *fli-1 *5' upstream region driving *gfp *(see Methods). Expression was observed in body wall muscle, pharyngeal muscle, and vulval muscle of embryonic, larval, and adult animals (Figure 9A). This is consistent with the Pat phenotype of *fli-1(tm362) *animals and the pharyngeal pumping defects of *unc-115; fli-1(ky535) *animals. In complex arrays (see Methods), expression was occasionally observed along the entire length the adult gonad in a "honeycomb" pattern characteristic of gonad expression (Figure 9B). This expression was faint and variable (not observed in all animals) and tended to dissipate as the complex array lines were maintained for more than three generations. This pattern could reflect expression in the germ line, the somatic sheath cells, or both. Male gonads, which are devoid of sheath cells, also showed faint and variable expression along their lengths (Figure 9B inset), suggesting that expression might be in the germ line. However, sheath cell expression cannot be excluded from these experiments.

The full-length *fli-1::gfp *transgene, predicted to encode a full-length FLI-1 polypeptide with GFP at the C-terminus, rescued *fli-1 *lethality and partially rescued the Glm phenotype of *fli-1(ky535) *and *fli-1(tm362)*, suggesting the FLI-1::GFP molecule was functional. No FLI-1::GFP fluorescence was detected in the gonads of these transgenic animals, and muscle expression was very faint and inconsistent. Possibly, FLI-1::GFP was expressed at very low levels, below detection in the gonad.

**Figure 10:**
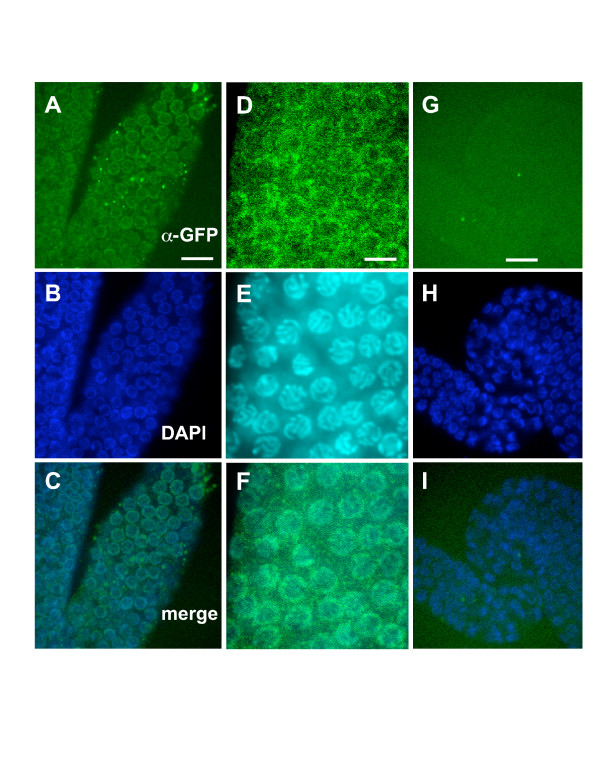
**FLI-1::GFP accumulates at germ line nuclei**. Confocal images of gonads from animals stained with anti-GFP antibody (green) and DAPI to label nuclei (blue). (A-F) are images of gonads from wild-type animals harboring the full-length *fli-1::gfp *transgene that rescues the *fli-1 *Glm phenotype and that is predicted to encode a full-length FLI-1 protein tagged with GFP at the C-terminus. (G-I) are images of a wild-type animal without the *fli-1::gfp *transgene. (A-C) are images of the transition zone (upper right) to pachytene zone (lower left); (D-F) are images of the pachytene zone. FLI-1::GFP reactivity was found associated with germ line nuclei. The scale bars in A and G represent 5 μm for A-C and G-I. The scale bar in D represents 2 μm for D-F.

To detect low levels of FLI-1::GFP expression, gonads from animals expressing full-length *fli-1::gfp *were excised and stained with an antibody against GFP. Specific GFP immunoreactivity was predominantly associated with germ nuclei (Figure 10A–F). Gonads from animals without the *fli-1::gfp *transgene showed no such reactivity (Figure 10G–I). FLI-1::GFP was associated with nuclei along the length of the entire gonad, and no obvious differences in FLI-1::GFP accumulation or nuclear association were detected along the length of the distal gonad from the mitotic zone through the meiotic zone.

### *let-60 *mutations display a germ line morphogenesis phenotype similar to *fli-1*

Previous studies described defects in germ line organization in mutants of Ras signaling pathway components: *mpk-1 *and *ksr-2 *mutations caused germ line clumping [17,29]; and *mek-2 *and *let-60 Ras *mutants displayed misplaced nuclei in the meiotic zone [16]. *C. elegans *LET-60 is similar to human k-Ras [30,31], and has been shown to control transition of germ nuclei from meiotic pachytene to diakinesis and germ line organization [16].

**Figure 11:**
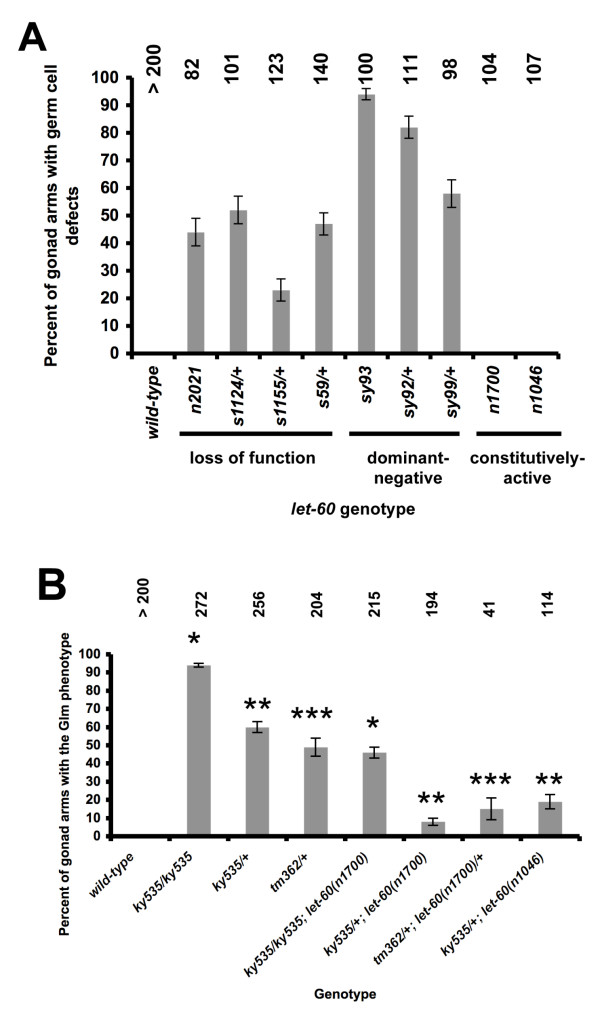
**Constitutively-active LET-60 suppresses the *fli-1 *Glm phenotype**. Genotypes are along the x axis, and percentage of gonad arms displaying the Glm phenotype are along the y-axis. Numbers of gonads scored for each genotype are indicated. Error bars represent the standard error of the proportion, and matching numbers of asterisks indicate that the genotypes are significantly different (t-test and Fisher's Exact analysis; p < 0.001). (A) Loss-of-function and dominant-negative alleles of *let-60 Ras *displayed the Glm phenotype whereas constitutively-active alleles did not. (B) Two constitutively-active alleles of *let-60 Ras *suppress the Glm phenotype of *fli-1(ky535) *and *fli-1(tm362)*/+.

To begin to characterize Ras signaling in the Glm phenotype, alleles of *let-60 Ras *that cause loss of function, constitutive activation, and dominant negative effects were analyzed for the germ line morphogenesis defect by DIC optics and DAPI staining (Figure 11A) [30,32,33]. The hypomorphic loss-of-function allele *n2021 *caused a *ky535*-like germ line defect in 44% of gonad arms, and the stronger *let-60 *loss-of-function alleles *s1124, s1155*, and *s59*, which are homozygous lethal, caused the Glm phenotype when heterozygous (52%, 23%, and 47%, respectively). These data suggest that *let-60 *is haploinsufficient for the Glm phenotype as was *fli-1*. Three different dominant-negative alleles of *let-60 *also displayed the germ line phenotype as homozygotes or as heterozygotes (e.g. 94% for homozygous *sy93*) (Figure 11A). TEM sections of *let-60(n2021) *showed a similar ultrastructural defect as *fli-1(ky535) *(data not shown), including germ line plasma membrane and sheath cell protrusions between misplaced nuclei. Furthermore, *let-60 *loss-of-function and dominant negative mutants displayed sheath cell *lim-7::gfp *expression associated with misplaced nuclei (Figure 5E, F, and 5I). While loss-of-function and dominant negative *let-60 *alleles caused the Glm phenotype, constitutively-active *let-60 *alleles *n1700 *and *n1046 *caused little or no Glm phenotype (Figure 11A).

### LET-60 activity can compensate for loss of FLI-1 in germ line morphogenesis

*fli-1 *and *let-60 Ras *mutations cause the Glm defect, and constitutively-active *let-60 *alleles, which presumably cause *let-60 *overactivation, had no apparent effect on germ line morphogenesis. The constitutively-active *let-60(n1700) *mutation partially suppressed the Glm defect of *fli-1(ky535) *heterozygotes and homozygotes and *fli-1(tm362)*/+ heterozygotes (Figure 11B). For example, *fli-1(tm362) *heterozygotes displayed 49% defective gonad arms, reduced to 15% by heterozygous *let-60(n1700)*. *let-60(n1046)*, another constitutively-active *let-60 *mutation, suppressed the Glm phenotype of *fli-1(ky535)/+ *heterozygotes (60% versus 18%). These data indicate LET-60 Ras overactivation can partially compensate for loss of *fli-1 *function and suggest that *fli-1 *and *let-60 Ras *act together to control germ line morphogenesis. Possibly, FLI-1 and LET-60 act in the same pathway or in parallel pathways to control germ line morphogenesis. It is also possible that FLI-1 and LET-60 control each others' expression.

## Discussion

Experiments described here show that mutations in *fli-1 *and *let-60 Ras *affect morphogenesis of the germ line (the Glm phenotype). *fli-1 *can encode an actin-binding protein similar to Drosophila and human Flightless-1 and has been shown to interact physically with human Ha-Ras via the leucine-rich repeats [20]. Studies described here show that overactivity of *let-60 Ras *can compensate for *fli-1 *loss-of-function in the germ line, suggesting that FLI-1 and LET-60 Ras act together to control germ line morphogenesis.

### The Glm phenotype

Meiotic nuclei are associated with the cortex of the germ line, forming the rachis. Sheath cell filopodia protrude superficially in the gaps between germ line plasma membrane that partially surround meiotic nuclei, but they do not protrude deeply [4]. *fli-1 *mutants displayed interconnected chains of meiotic nuclei spanning the rachis of the meiotic zone, a configuration never seen in wild-type. These misplaced nuclei were partially surrounded by invaginations of germ line plasma membrane as were their normally-positioned counterparts at the cortex. Gonadal sheath cell projections protruded between the plasma membrane invaginations and were in close proximity to the nuclei deep in the center of the rachis region (see Figure 6 for a diagram of these results). Such deep sheath cell projections in the meiotic zone were never observed in wild type. In *fli-1 *mutants, the projections between misplaced nuclei in the meiotic zone were not from the distal tip cell, but were from the more proximally-located sheath cells that normally do not protrude deeply between nuclei.

Misplaced chains of nuclei in *fli-1 *mutants were not due to defects in meiotic progression, as all aspects of meiosis appeared normal in *fli-1 *mutants: nuclei in the meiotic zone did not incorporate BrdU, suggesting that they were post-mitotic; the morphology of misplaced nuclei was similar to normal meiotic nuclei as judged by DAPI staining and by electron microscopy. Thus, while *fli-1 *mutant germ nuclei apparently underwent meiosis normally, they failed to organize properly to form the rachis.

Data presented here suggest that *fli-1 *affects germ line morphogenesis without affecting meiotic progression or other aspects of germ line differentiation. However, *fli-1(ky535) *is a hypomorph and *fli-1(tm362) *homozygotes arrested in embryogenesis before germ line development (the germ line phenotype was scored in *fli-1(tm362) *heterozygotes). All *fli-1 *genotypes in which the Glm phenotype was scored had some *fli-1 *activity, so it is possible that complete loss of *fli-1 *activity in the germ line would affect other aspects germ line development not apparent in these studies (e.g. meiotic progression to diakinesis similar to *let-60 Ras *or other aspects of meiotic differentiation). Possibly, FLI-1 is required for the proper formation or maintenance of the rachis through effects on the actin cytoskeleton or the germline plasma membrane. Alternatively, FLI-1 might be part of a developmental program that coordinates rachis formation with other aspects of meiotic differentiation.

### The Glm phenotype might be sensitive to gene dosage

Animals heterozygous for both *fli-1(ky535) *and *fli-1(tm362) *displayed the Glm phenotype. *let-60 Ras *was also haploinsufficient, as heterozygous *let-60 Ras *loss-of-function mutations displayed the Glm phenotype. These data suggest that precise dosages of FLI-1 and LET-60 Ras are required for normal germ line morphogenesis, and that reduction by as little as one half can cause the Glm phenotype. It is also possible that *ky535 *and *tm362 *are not simple loss-of-function alleles and that each might have a gain-of-function effect, explaining the Glm defect of heterozygous animals. In any case, RNAi of *fli-1 *results in the Glm phenotype, suggesting that the Glm phenotype is a consequence of loss of *fli-1 *function.

No nucleotide lesion associated with *ky535 *was detected in the region that can rescue the *fli-1(ky535) *and *fli-1(tm362) *Glm phenotypes. However, *ky535 *was mapped genetically to the *fli-1 *region using the Glm phenotype, and *fli-1 *RNAi phenocopied the *ky535 *phenotype. Furthermore, the Glm phenotype of *ky535 *was rescued by a *fli-1(+) *transgene containing only the *fli-1 *gene. Possibly, *ky535 *is a mutation outside of the rescuing region that reduces but does not abolish *fli-1 *expression, such as a mutation in a distal enhancer element. The haploinsufficiency of the *fli-1 *locus is consistent with this idea. The *fli-1(tm362) *deletion also displayed a haploinsufficient Glm phenotype rescued by a *fli-1(+) *transgene.

### FLI-1 and LET-60 Ras might act together to control germ line morphogenesis

*let-60 Ras *loss-of-function and dominant-negative mutations caused the Glm phenotype similar to *fli-1*. Constitutively-active alleles of *let-60 Ras *did not. Previous studies showed a germ cell organization defect in *let-60 *and *mpk-1 *mutants [16,17]. *mpk-1 *caused large clumps of nuclei with regions of the germline barren of nuclei, a defect rarely seen in the *fli-1 *and *let-60 *analyses described here. Possibly, the defects of *fli-1 *and *mpk-1 *are related, and *mpk-1 *has a stronger effect than *fli-1*. Alternatively, *fli-1 *and *mpk-1 *might affect distinct processes.

Interestingly, the Glm phenotype of *fli-1 *mutations was suppressed by constitutively-active *let-60 Ras *mutations, suggesting that LET-60 Ras overactivity compensated for FLI-1 loss of function. In these experiments, FLI-1 activity was reduced but not eliminated (*ky535 *is a hypomorph, and *tm362 *was heterozygous). Thus, it is possible that LET-60 Ras and FLI-1 act together in the same pathway or in parallel pathways to control germ line morphogenesis. The LRRs of *C. elegans *FLI-1 interact physically with human Ha-Ras in vitro [20] suggesting that FLI-1 and Ras might act in the same pathway. Another possibility is that *let-60 *controls *fli-1 *expression. Indeed, microarray expression analysis indicates that *fli-1 *transcript levels are increased by constitutively-active *let-60(G12V) *[34]. Further experiments will be required to test these models of FLI-1 and LET-60 interaction.

### FLI-1 is expressed in the gonad

The *fli-1 *promoter was active in muscle cells and in the gonad. Anti-GFP Immunofluorescence revealed that FLI-1::GFP was associated with germ line nuclei. The expression pattern of *fli-1 *is consistent with expression in the germ line, but expression in the somatic sheath cells cannot be excluded. Furthermore, rescue of the *fli-1(ky535) *and *fli-1(tm362) *Glm phenotype could be due to somatic or germline transgene expression. FLI-1 might be expressed and active in the germ line, in the somatic sheath cells, or both. RNAi of *fli-1 *in *rrf-1 *mutants led to the Glm phenocopy, suggesting that knock-down of *fli-1 *in the germ line causes the Glm phenotype. However, *fli-1 *is very sensitive to gene dosage, so even slight perturbation of *fli-1 *in the soma of *rrf-1 *animals might be enough to cause the phenotype. *fli-1 *males also showed the Glm phenotype, and male gonads do not have somatic sheath cells. Together, these data suggest that *fli-1 *acts in the germ line, but they do not exclude the possibility that *fli-1 *acts in the sheath cells or in another tissue.

Human Fliih acts in the nucleus as a component of a coactivator complex and with the TCF/LEF and β-catenin complex [22,23]. However, Fliih also associates with microtubule- and actin-based structures in the cytoplasm of fibroblasts, and acts with small GTPase and PI3 kinase signaling in the cytoplasm [35]. It is unclear from these experiments if FLI-1 acts in the nucleus or cytoplasm in germ line morphogenesis. FLI-1 could act in the nucleus to regulate expression along with Ras signaling. Alternatively, FLI-1 could act in the cytoplasm in a pathway parallel to a transcriptionally-dependent Ras pathway, possibly by modulating cytoskeletal architecture involved in germ line reorganization, although no defects in germ line actin organization were apparent. Further studies will address these models of molecular mechanisms of FLI-1 and LET-60 Ras function in germ line morphogenesis.

## Conclusion

This work describes the role of the FLI-1 molecule in germ line morphogenesis in *C. elegans*. While much is known about meiotic differentiation in *C. elegans*, less is known about the mechanisms that control meiotic germ cells organization at the periphery of the germ line to form a germ cell-free core of cytoplasm called the rachis. Mutations in *fli-1 *perturb rachis organization without perturbing meiotic differentiation. In *fli-1*, germ cell nuclei occupied positions in the rachis; these misplaced nuclei were partially enclosed by germ line plasma membrane as were nuclei at the cortex; and extension of the gonadal sheath cells were associated with misplaced nuclei deep in the rachis. Mutations in *let-60 Ras *also displayed this phenotype, and constitutively-active LET-60 partially compensated for loss of FLI-1, indicating that LET-60 Ras and FLI-1 might act together to control germ line morphogenesis. These studies describe a developmental role for the FLI-1 molecule in germ line morphogenesis and demonstrate a functional interaction between FLI-1 and Ras GTPases in this process.

## Methods

### *C. elegans *strains and genetics

*C. elegans *were cultured by standard techniques [36,37]. All experiments were done at 20°C unless otherwise noted. The Bristol strain N2 was used as the wild-type. The following mutations and transgenes were used. LGX: *unc-115(mn481), sem-5(n2089)*. LGI: *mek-2(n1989), sur-2(ku9)*. LGII: *let-23(n1045), let-23(sy10), lin-31(n301)*. LGIII: *fli-1(ky535)*, *fli-1(tm362)*, *tnIs6 [plim-7::gfp]*, *dpy-17(e164), unc-32(e189), mpk-1(ku1), eT1*. LGIV: *let-60(n2021)*, *let-60(s1124)*, *let-60(s1155)*, *let-60(s59)*, *let-60(sy93)*, *let-60(sy92)*, *let-60(sy99)*, *let-60(n1046)*, *let-60(n1700)*, *lin-3(e1417)*, *lin-3(n1058), lin-1(n431)*. LGV: *sos-1(s1031), lin-25(e1446), qIs56 [lag-2::gfp]*.

Transgenic *C. elegans *were produced by germ line microinjection of DNA solutions using standard techniques [38]. Cosmid DNAs were injected at 100 ng/μl, and *fli-1 *fragments generated by PCR were injected at 25 ng/μl. To visualize germ line expression of *fli-1 *transgenes, complex arrays were constructed using fragmented *C. elegans *genomic DNA in the injection mix [39]. *fli-1::gfp *expression in the germ line was unstable and became non-visible as the transgenes were propagated. For the *fli-1::gfp *immunofluorescence experiments, new complex-array transgenic lines were produced before each experiment to ensure robust *fli-1::gfp *expression in the germ line.

The germ line morphogenesis phenotype (Glm) was quantitated by scoring the percentage of gonad arms that displayed chains of nuclei spanning the rachis of the meiotic pachytene zone. In wild-type, chains of nuclei were often observed in the transition zone where reorganization occurs. Care was taken to ensure that the Glm phenotype was scored clearly in the meiotic pachytene zone and not in the transition zone. Significance of quantitative data was determined by the t-test and by Fisher's Exact analysis (for percentages).

### *fli-1 *molecular biology

Fragments of the *fli-1 *gene were amplified using polymerase chain reaction (PCR). The sequence of all coding regions generated by PCR were determined to ensure that no errors were introduced. The *fli-1 *whole gene consisted of bases 8,674,953–8,684,714 of linkage group III. For *fli-1(ky535) *sequencing, this region was amplified in three overlapping fragments and their sequences determined. In three separate amplifications, no nucleotide changes were detected in *fli-1(ky535) *DNA. The full-length *fli-1::gfp *transgene was produced by amplifying a region including the *fli-1 *upstream and *fli-1 *coding region but not including the stop codon or downstream region (bases 8,676,004–8,684,714 linkage group III). This fragment was then fused in-frame to *gfp *in vector pPD95.77 (kindly provided by A. Fire). The *fli-1 promoter::gfp *fusion was produced by amplifying the *fli-1 *upstream region (8,683,600–8,684,714 of linkage group III) and fusing the fragment upstream of *gfp *in pPD95.77. RNA-mediated gene interference (RNAi) was performed by microinjection of double-stranded RNA [40], representing a portion of *fli-1 *exon 6 (see Figure 4), into the germ line and analyzing the germ line phenotype in progeny of injected animals. The sequences of all oligonucleotide primers used in this study are available upon request.

### Imaging and microscopy

Differential Interference Contrast (DIC) and epifluorescence images were taken using a Leica DMR light microscope with a Hamamatsu Orca camera. Some images were obtained with an Olympus spinning disk confocal microscope. For electron microscopy, samples were examined and photographed using a JEOL1200EXII transmission electron microscope and a MegaView camera (Soft Imaging System). Images were adjusted for contrast, cropped, and overlayed using Adobe Photoshop.

### TEM specimen preparation and analysis

Hermaphrodites (12 hours after the L4/adult molt) were fixed using a modification of the procedure previously described [41]. Worms were anaesthetized immersing them in 8% EtOH in M9 buffer for 5 minutes and were fixed by immersion in 2.5% glutaraldehyde, 1% formaldehyde in 0.1 M sucrose, 0.05 M Na-cacodylate, pH7.4, for 30 min at 4°C. Animals then were cut in half using a scalpel and returned to the fixative and incubated overnight at 4°C. They then were rinsed 3 times (10 minutes each) at 4°C with 0.2 M Na-cacodylate, pH 7.4, and then post-fixed for 90 minutes, at 4°C, with 0.5% OsO_4_, 0.5% KFe(CN)_6 _and 0.1 M Na-cacodylate, pH 7.4, for 90 minutes on ice. Subsequent steps were carried out at room temperature. Worms then were rinsed three times, 10 minutes each, in 0.1 M Na-cacodylate buffer, stained in 1% uranyl acetate in 0.1 M sodium acetate, pH 5.2, for 1 hour at room temperature, followed by three 5-minute 0.1 M sodium acetate washes and three 5-minute distilled water washes. Worms were packed in parallel in a V-shaped plexiglass trough and were embedded 3% seaplaque agarose. Approximately 1 mm^2 ^blocks then were dehydrated in acetone and embedded in Embed 812 [42].

For each genotype examined, at least three individual animals were sectioned, and multiple sections from each animal along the entire gonad span were analyzed. Cross-sections of worms were cut using a diamond knife and Leica microtome and were picked up on carbon-over-formvar coated single hole grids. Sections were dried overnight and then stained using minor modifications of the Hall (1995) procedure. Stains and washes were prepared in 16 well plastic culture dishes at room temperature. Grids were stained in 1% uranyl acetate, 50% methanol for 15 minutes, rinsed twice (30 seconds each) with 100% ethanol followed by 50% ethanol/water (15 seconds), 30% ethanol/water (15 seconds), and four 15 second washes in water. Sections then were stained for 5 minutes with 0.1% lead citrate in 0.1 M NaOH, rinsed twice with 0.02 M NaOH (1 minute/change), rinsed five times in water (15 seconds/wash) and were air-dried before examination with the TEM.

### DAPI and phalloidin staining of dissected gonads

The gonads of 12-hour-old adult hermaphrodite animals were dissected and fixed with 3% paraformaldehyde containing 0.1 M K_2_HPO_4_, pH7.2, for 1 hour at room temperature. The specimens were washed once with phosphate-buffered saline (PBS) with 0.1% Tween-20 (PBT) for 5 minutes followed by treatment with 100% methanol for 5 minute at -20°C. Specimens were treated with PBS containing 100 ng/μl 4',6-diamidino-2-phenylindole (DAPI) or rhodamine-phalloidin for 10 minutes at room temperature followed by three washes in PBT. Gonads were mounted on a 2% agarose pad in M9 buffer with 1 mg/ml 1,4-diazabicyclo [2.2.2]octane (DABCO) antifade reagent.

### BrdU labeling of dissected gonads

*Escherichia coli *strain MG1693 (a thymidine-deficient *E. coli *strain kindly provided by the *E. coli *stock center) were grown minimal medium (M9) with 0.4% glucose, 1 mM MgSO_4_, 1.25 μg/ml vitamin B1, 0.5 μM thymidine, and 10 μM bromodeoxyuridine (BrdU) overnight at 37°C [25]. BrdU-labeled *E. coli *were then plated on nematode growth medium (NGM) plates containing 100 μg/ml ampicillin. 12-hour-old adult hermaphrodite animals were placed on seeded plates and allowed to eat the BrdU-labeled *E. coli *for varying times depending on the experiment (usually 5 minutes). Gonads were dissected immediately and fixed in methanol at -20°C for 1 hour followed by 1% paraformaldehyde for 15 minutes at room temperature.

Fixed gonads were placed in 1 mg/ml BSA in PBT for 15 minutes, 2N HCl to denature DNA for 30 minutes at room temperature, and 0.1 M sodium borate to neutralize for 15 minutes at room temperature. The specimens were blocked in 1 mg/ml BSA in PBT for 15 minutes and stained with a 1:2.5 dilution in PBT of anti-BrdU antibody (B44, Becton-Dickinson, San Jose, CA) at 4°C overnight. On the next day, the specimens were washed three times by 1 mg/ml BSA in PBT for 10 minutes each. A 1:500 dilution of Alexa 488-conjugated goat-anti-mouse antibody was incubated with the specimens at room temperature for 2 hours in PBT. The specimens were washed three times with 1 mg/ml BSA in PBT for 10 minutes each with DAPI in the last wash to stain DNA (see above). Gonads were mounted for microscopy as described above.

### Anti-GFP immunofluorescence of dissected gonads

Gonads of adult hermaphrodite animals (12 hours after the L4/adult molt) were fixed as described for DAPI staining. Fixed gonads were blocked for 1 hour in 1 mg/ml BSA in PBT at room temperature and then were incubated overnight with 1:50 diluted monoclonal anti-GFP (Sigma-Aldrich, St. Louis, MO) antibody at 4°C. The specimens were washed three times with PBT for 10 minutes each and incubated with 1:500 diluted Alexa 488-conjugated goat-anti-mouse antibody (Sigma-Aldrich, St. Louis, MO) for 2 hours at room temperature. DAPI was included to stain DNA. Stained gonads were rinsed three times with PBT for 10 minutes each. Gonads were mounted for microscopy as described above.

## Authors' contributions

JL conducted all of the experiments described in the manuscript and participated in figure construction and manuscript writing. WLD assisted with transmission electron microscopy and discussion of results. EAL oversaw all aspects of the project and contributed the bulk of manuscript writing.
